# Red Cell Distribution Width Is Positively Correlated with Atherosclerotic Cardiovascular Disease 10-Year Risk Score, Age, and CRP in Spondyloarthritis with Axial or Peripheral Disease

**DOI:** 10.1155/2018/2476239

**Published:** 2018-09-27

**Authors:** Hassan Ahmad, Mariam Khan, Michelle Laugle, Desmond A. Jackson, Christopher Burant, Charles J. Malemud, Ali D. Askari, Maya Mattar, David E. Blumenthal, David A. Zidar, Donald D. Anthony

**Affiliations:** ^1^Rheumatology Section, Louis Stokes Cleveland VA, Cleveland, OH, USA; ^2^Department of Medicine, Division of Rheumatic Diseases, Case Western Reserve University, Cleveland, OH, USA; ^3^Division of Rheumatic Diseases, University Hospitals Cleveland Medical Center, Cleveland, OH, USA; ^4^Cleveland VA Geriatric Research, Education and Clinical Center (GRECC), USA; ^5^Harrington Heart & Vascular Institute, University Hospitals Cleveland Medical Center, Louis Stokes Cleveland VA, Cleveland, OH, USA

## Abstract

**Background:**

Red blood cell distribution width (RDW) is a routine hematologic parameter that is a predictor of cardiovascular disease (CVD) events and is independent of combined traditional risk factor scoring systems. The RDW has also been associated with rheumatic disease activity. Whether RDW is associated with traditional CVD risk factors or Atherosclerotic Cardiovascular Disease (ASCVD) 10-year CVD risk score in patients with seronegative spondyloarthritis with axial or peripheral disease has not been previously determined.

**Methods:**

We performed a retrospective, chart review study evaluating the relationship between RDW, albumin, hemoglobin, C-reactive protein (CRP), absolute lymphocyte count (ALC), and ASCVD scoring parameters [age, hypertension status, diabetes mellitus (DM) status, lipid profile, and smoking status] in a cohort of spondyloarthritis patients, taking into consideration their HLA-B27 status, race, and treatment status.

**Results:**

RDW was found to positively correlate with ASCVD 10-year score and age, and ASCVD score did not change over time after patients were treated for spondyloarthritis. Albumin was found to negatively correlate with ASCVD 10-year risk score. Both RDW and albumin correlated with CRP. ALC failed to correlate with ASCVD 10-year score but did show a tendency to be associated with CVD, CVD events, and cardiac conduction abnormalities.

**Conclusions:**

These data indicate that further study is warranted to evaluate RDW, albumin level, and ALC as potential predictors of CVD in the spondyloarthritis patient population.

## 1. Introduction

Ankylosing Spondylitis (AS) is a chronic systemic inflammatory disease affecting the sacroiliac joints, spine, and peripheral joints [1]. Cardiac involvement has also been reported in 2-10% of patients with AS [2]. The most common cardiac manifestations are aortic root, aortic valve, and cardiac conduction abnormalities at the atrioventricular (AV) node [3, 4], whereas diastolic dysfunction appears to be the predominant myocardial dysfunction in AS patients [5, 6]. In the general non-AS population, men with pacemakers are known to have significantly increased frequency of HLA-B27 expression compared to other HLA class 1 haplotypes [7]. Furthermore, both cardiac conduction abnormalities and aortic regurgitation have been observed in patients with HLA-B27-related extracardiac manifestations, regardless of their AS- arthritis severity [7]. The incidence and prevalence of coronary artery disease (CAD) in AS is less well described, although a 2-3-fold increase in the rate of myocardial ischemia (MI) in AS patients (4.4%) compared to the general population has been reported [8].

The 2013 American College of Cardiology/American Heart Association (ACC/AHA) cardiovascular (CV) risk score calculates a 10-year Atherosclerotic Cardiovascular Disease (ASCVD) risk based on pooled cohort risk equation [9]. Compared to the older cardiovascular risk assessment algorithms such as the Framingham Risk Score (FRS), the newer algorithm includes a patient's 10-year risk of stroke in addition to coronary events, uses separate sex- and race-specific equations, and also allows clinicians to estimate lifetime risk for cardiac-related events. The 2013 ACC/AHA ASCVD 10-year risk score is used to identify high-risk individuals who could benefit from statin therapy. For example, in the setting of RA, the ASCVD 10-year score was better correlated with high coronary artery calcium (CAC) scores when compared to the FRS and Reynolds risk score (RRS) [10]. Given other nontraditional inflammatory mechanisms are likely involved in the setting of autoimmunity, identifying additional biomarkers beyond information conveyed by the ASCVD 10-year score may be clinically important.

The higher risk of CVD in patients with autoimmune rheumatic diseases has been attributed to traditional cardiovascular risk factors as well as chronic systemic inflammation [11]. It has been postulated that increased red blood cell distribution width (RDW) reflects underlying chronic inflammation which contributes to the increased risk of CVD [12]. In fact, it has been reported that increased RDW is strongly and independently associated with the risk of cardiovascular morbidity and mortality in patients with a history of MI [12]. RDW positively correlates with inflammatory markers such as C-reactive protein (CRP) and erythrocyte sedimentation rate (ESR) even after excluding anemia [13]. In fact, RDW has been proposed to be an inflammatory marker, and the association between RDW and CVD has been proposed to be partially mediated by an inflammatory response. It is plausible that RDW is a suitable biomarker for estimating the activity of autoimmune disease and cardiovascular risk. However, there are few reported studies investigating RDW in the setting of AS or spondyloarthritis with axial or peripheral disease. The results of two studies indicated that RDW is increased in the setting of AS [5, 14]. Furthermore, RDW positively correlates with the AS disease activity as measured by Bath Ankylosing Spondylitis Disease Activity Index (BASDAI) [5, 14]. Given that RDW is both associated with CVD and autoimmune disease activity, further investigation of RDW in relation to CVD parameters in the setting of spondyloarthritis may fill an important gap in knowledge.

## 2. Methods

### 2.1. Study Population

This retrospective chart review study was approved by the Institutional Review Board of the Cleveland Louis Stokes Veteran's Administration Medical Center (VAMC). All VAMC electronic medical records were reviewed for HLA-B27 testing. Six hundred forty-one (641) adult patients (greater than 18 years of age) who had HLA-B27 testing at the VAMC between 2007 and 2017 were identified. Of these, 95 patients were verified to have an ICD-9/10 diagnosis of AS. We reviewed the patient's charts to determine who fulfilled the 2009 ASAS criteria for the diagnosis of spondyloarthritis with axial or peripheral disease [15]. Those charts that strictly met the ASAS criteria for a diagnosis of spondyloarthritis with high confidence (n=49) were further reviewed. We included the individual parameters from July 1, 2013, to July 1, 2017, in order to calculate the most recent data for ASCVD 10-year risk assessment. The only exception was the pretreatment RDW value, which was counted from any time in the VAMC records confirmed to be prior to initiation of treatment of spondyloarthritis. Six patients were excluded due to either failure to follow up with a rheumatology assessment and/or if patients did not have adequate data on ASCVD parameters. Forty-three patients were classified as having analyzable lipid profile laboratory studies and a diagnosis of spondyloarthritis with axial and/or peripheral disease based on the ASAS criteria.

### 2.2. Data Extraction

Charts were reviewed for lipid profiles (high density lipoproteins and total cholesterol), hemoglobin A1c (HbA1c), smoking history, diagnosis of hypertension (HTN), systolic blood pressure (SBP), antihypertensive usage, diagnosis of CVD, diagnosis of diabetes mellitus (DM), diagnosis of hyperlipidemia, RDW, CRP, hemoglobin, absolute lymphocyte count (ALC), albumin level, treatment with nonsteroidal anti-inflammatory drugs (NSAIDs), treatment with conventional synthetic disease-modifying antirheumatic drugs (csDMARDs), including methotrexate, sulfasalazine, and leflunomide, treatment with biologic monotherapy (including tumor necrosis factor inhibitors (TNF-I), interleukin-17 antagonists (IL-17A)), and combination biologic and csDMARD therapy. For laboratory values, at least two data points that were found within six months of when the lipid profile was taken were obtained, except in the case of pretreatment RDW. A patient was considered to be on treatment if there was documentation of the medication prescription including refills on the VAMC pharmacy tracking page during the chart review period used to calculate the ASCVD 10-year risk score. The pretreatment time period was identified by both reviewing pharmacy records for absence of these spondyloarthritis class medications and review of the rheumatology notes to confirm that no outside medications were being prescribed.

### 2.3. ASCVD 10-Year Risk Score

The ASCVD 10-year risk score was calculated using the ACC/AHA 2013 criteria [16]. For patients less than 40 years of age, we imputed age to 40 for purposes of calculating the ASCVD 10 year risk score.

### 2.4. Statistical Analysis

Statistical analyses were performed using SPSS for Windows v. 24.0 (IBM Corp, Armonk, New York). Correlations between continuous variables were evaluated by calculating the Spearman rank correlation coefficient. Group comparisons were analyzed by the Mann-Whitney U test. All tests of significance were two-sided and p values of ≤ 0.05 were considered significant.

## 3. Results

Demographics and laboratory features of the 43 patients with spondyloarthritis are shown in Table 1. The cohort had a median age of 56 years (range 27-78). All patients, with the exception of 1, were male. The 10-year ASCVD risk score ranged from 0.7 to 62.2% (median of 13.1%). Thirty-two (74%) were HLA-B27 positive. As expected, those patients that were HLA-B27 negative were more likely to be African-American (p=0.008). Iritis was commonly diagnosed (30%), while inflammatory bowel disease and psoriasis were less common (14% and 2%, respectively). Five patients were noted to be treated with only csDMARDs, whereas 24 were on biologic monotherapy, and 6 were treated with a combination of csDMARDS and biologics.

**Figure 1:**
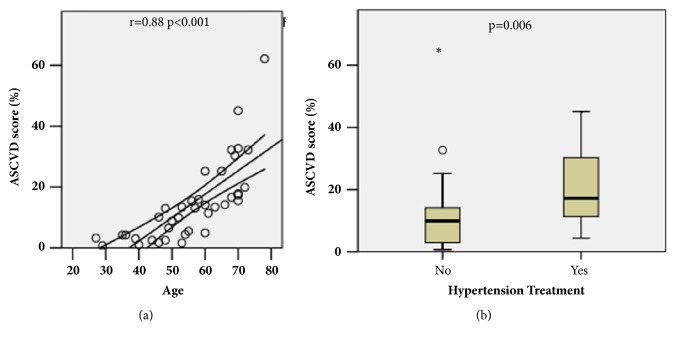
Age and hypertension treatment status scoring components are associated with ASCVD 10-year risk score. (a) Age (years) versus ASCVD 10-year risk score (%). Spearman's r and p values shown, along with linear trend line and 95% confidence intervals. (b) Box plot of hypertension treatment status versus ASCVD 10-year risk score (%). The p value was calculated by the Mann-Whitney U test.

We first examined how each of the parameters that compose the ASCVD 10-year risk score correlated with the ASCVD 10-year risk score itself in this patient population in order to identify which parameters were driving the score. The ASCVD 10-year risk score was positively correlated with age (r= 0.88, p<0.001, Figure 1(a)). The score was also greater in those treated for hypertension (p=0.006, Figure 1(b)). However, the ASCVD 10-year risk score did not correlate with cholesterol level (p=0.33), HDL level (p=0.66), SBP (p=0.30), smoking status (p=0.18), or race (p=0.42), although ASCVD score tended to be associated with statin use (p=0.06) and DM status (p=0.07). These data indicated that age and hypertension treatment status were the principal drivers of the ASCVD 10-year risk score in this spondyloarthritis cohort.

**Figure 2:**
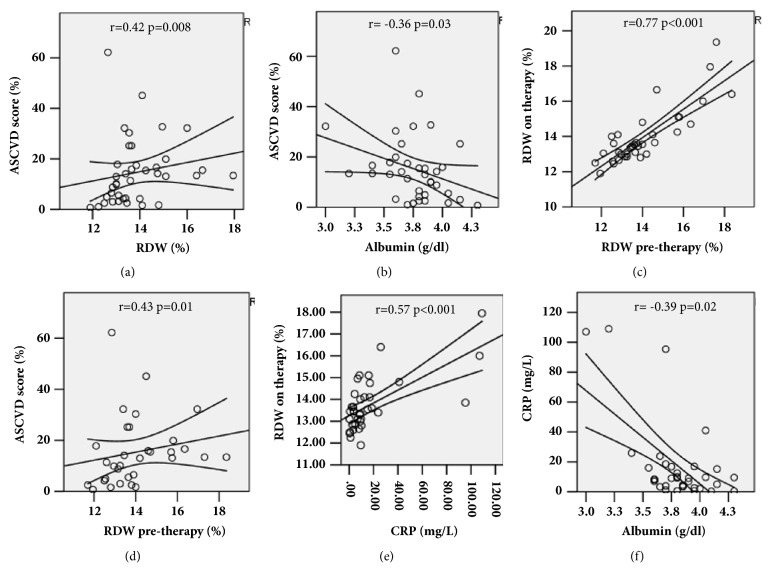
ASCVD 10-year risk score is correlated with RDW and albumin. (a) RDW versus ASCVD score %. (b) Albumin versus ASCVD score %. (c) RDW pretherapy versus on therapy. (d) RDW pretherapy versus ASCVD score %. (e) RDW versus CRP. (f) CRP and albumin. Spearman's r and p values are shown, along with the linear trend line and 95% confidence intervals.

We next examined whether other parameters were associated with ASCVD 10-year risk score in this cohort, with a particular focus on RDW. Albumin, hemoglobin, and ALC were also evaluated since these are biomarkers commonly followed in our patients with inflammatory disease, and HLA-B27 status was evaluated given its association with spondyloarthritis. We found that ASCVD score was positively correlated with RDW (r=0.42, p=0.008, Figure 2(a)), and negatively correlated with albumin (r= -0.36, p=0.03, Figure 2(b)). ASCVD was not correlated with ALC (p=0.8), hemoglobin (p=0.2), or HLA-B27 status (p=0.46) (data not shown).

To identify whether the relationship between RDW and ASCVD 10-year risk score was influenced by therapy or the clinical control of spondyloarthritis, we also examined pretreatment RDW, and whether this value differed from treated spondyloarthritis RDW or was itself correlated with ASCVD 10-year risk score. We observed no significant change in RDW when comparing pretreatment to “on therapy” values (p=0.31), and in fact, pretreatment RDW was closely correlated with “on therapy” RDW (r=0.77, p<0.001, Figure 2(c)). Furthermore, pretreatment RDW was correlated with ASCVD 10-year risk score (r=0.43, p=0.01, Figure 2(d)). To further evaluate whether treatment status influenced ASCVD 10-year risk score or RDW, we examined whether ASCVD 10-year risk score and RDW differed between those treated and not treated with csDMARDs, biologics, or combination therapy. Of note, ASCVD score was not associated with csDMARD treatment status (p=0.7), biologic treatment status (p=0.7), or combination csDMARD plus biologic treatment status (p=0.5). Similarly, albumin failed to correlate with any of these treatments (csDMARDS, p= 0.5, biologics, p=0.6; csDMARDs plus biologics, p=0.5). Finally, although ASCVD 10-year risk score was not associated with CRP (p=0.89), RDW and albumin were associated with CRP (r=0.57 p<0.001, r= -0.39 p=0.02, Figures 2(e) and 2(f)). These data indicated that traditional CVD risk factors, as reflected by ASCVD 10-year risk score, were associated with RDW and albumin, whereas RDW and albumin, but not ASCVD 10-year risk score, were also associated with CRP.

To better understand which parameters were the potential drivers of RDW and albumin levels we evaluated relationships between RDW, albumin, and other clinical parameters. In addition to RDW correlating with ASCVD 10-year risk score and CRP, RDW also correlated with age (r=0.360, p=0.016), hemoglobin (r= -0.577, p<0.001), and albumin (r= -0.437, p=0.004) and was associated with HLA-B27 status (p=0.018), race (p=0.048), and statin use (p=0.038). In addition to albumin correlating with ASCVD 10-year risk score, RDW, and CRP, albumin also correlated with age (r= -0.34, p=0.03) and hemoglobin (r=0.41, p=0.007). These data indicated that age may be a common and dominant driver of the ASCVD 10-year risk score, RDW, and albumin, whereas CRP, race, and B27 status were additional potential drivers of RDW.

**Figure 3:**
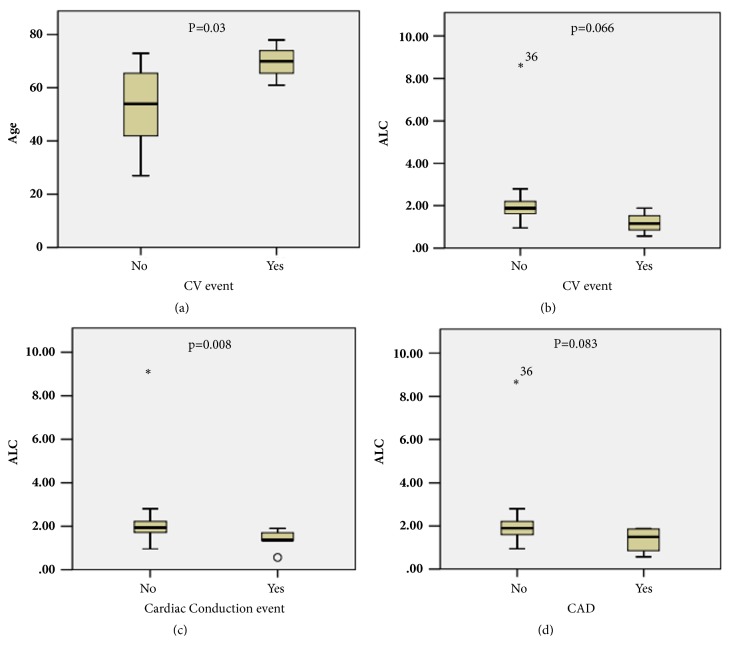
Age and ALC correlations with CVD. (a) Age is associated with CVD events. (b) ALC is nearly associated with CVD events. (c) ALC is significantly associated with conduction abnormalities. (d) ALC is nearly correlated with CAD.

Although our sample size is relatively small, 7 patients had cardiac conduction abnormalities (4 with atrial fibrillation, 2 with supraventricular tachycardia, and 1 with Wolf-Parkinson White), 4 had a confirmed diagnosis of CAD, and 3 had CVD events. Age was greater in those with a CV event (p=0.03, Figure 3(a)). ALC was nearly lower in those with a CV event (p=0.066, Figure 3(b)) and a diagnosis of CAD (p=0.08, Figure 3(d)) and was lower in those with a cardiac conduction abnormality (p=0.008, Figure 3(c)).

## 4. Discussion

It is known that autoimmune rheumatic diseases such as RA and SLE have increased cardiovascular morbidity and mortality compared to the general population [17]. The greater relative risk has been attributed to traditional cardiovascular risk factors as well as chronic systemic inflammation [11]. In the setting of RA and SLE, one cross-sectional study also identified the discordance between the CV risk assessment using 2013 ACC/AHA 10-year risk score (ASCVD 10-year risk score) versus the FRS and a modified FRS (with a 1.5 multiplier recommended by European League Against Rheumatism (EULAR) to capture the increased CV risk in RA patients) [18]. Specifically, 10% of SLE and RA patients had discordant 10-year CV risk scores with high 2013 ACC/AHA 10-year risk scores and low FRS, even when it was modified by a 1.5 multiplier [18]. This finding highlights the need for better CVD predictive measures in autoimmune patient populations. In the setting of spondyloarthritis with axial or peripheral involvement, conduction and structural abnormalities have been reported, although there is limited understanding regarding their relationship with atherosclerotic disease risk [3–6, 8]. Therefore, one way to better understand traditional factors associated CVD risk in AS is to evaluate the ASCVD 10-year risk score.

In this retrospective chart study, ASCVD 10-year risk score ranged from 0.7 to 62.2% with a median of 13.1% in a middle aged (median 56, range 27-78) male VA patient population. When evaluating drivers of the ASCVD 10-year risk score we found age and hypertension treatment status were strongly associated with the ASCVD 10-year risk score, whereas the other ASCVD risk score parameters were not. A recent retrospective, cross-sectional Dutch study estimated the 10-year CV risk in AS patients according to CV risk algorithms used in the Dutch, European, and American CV-RM guidelines (2013 ACC/AHA 10-year risk) [14]. The results of this study found substantially higher rates of hypertension (41% versus 31%) and smoking (43% versus 27%) in AS patients as compared to the general Dutch population, where 37% of these patients had an indication for CV risk treatment, thus illustrating the importance of cardiovascular risk management in AS patients.

RDW has been proposed to be a useful parameter for evaluating both CVD risk and disease activity during autoimmune disease. RDW is a measured index of the heterogeneity of the erythrocytes which reflects variability in the size of circulating RBCs [13]. Conditions associated with bone marrow dysfunction such as inflammation and/or erythropoietin resistance or that cause more immature cells to be released into the bloodstream, including abnormal hemoglobin, hemolysis (as in hemolytic anemias), can modify the shape of RBCs, resulting in an increased RDW [19]. In the setting of RA, RDW has been found to be higher when compared to osteoarthritis, and RDW was positively correlated with CRP regardless of anemia [20]. RDW was also positively correlated with DAS-28 disease activity total for RA [9, 17]. Moreover, increased RDW in RA patients was reported to be associated with the greater risk of cardiovascular diseases/cardiovascular events (i.e., heart failure, ischemic heart disease, or cerebrovascular incident), and this correlation remained significant even after adjusting for sex and gender [9]. Higher RDW has also been reported in the setting of SLE [7], and therapeutic outcomes have been negatively associated with RDW [16]. Our study in spondyloarthritis patients indicates that RDW correlates with both age and the ASCVD 10-year risk score. However, we also found that the RDW does not significantly change after therapy for spondyloarthritis. RDW was also associated with CRP, hemoglobin, albumin, HLAB27, race, and statin use. With respect to age, Shiga et al. demonstrated that RDW correlated with age in both genders [21]. Nevertheless, RDW is predictive of CVD independent of age in large nonrheumatic disease populations [21]. Thus, additional study is warranted to determine whether RDW is associated with clinical CVD events and, if so, whether or not this is related to traditional risk factors, such as those reflected in the ASCVD risk score.

Our study was limited by its retrospective nature and small sample size. Although we used an average of two RDW values in close proximity to the lipid profile that was employed to calculate the ASCVD 10-year risk score (no value more than 6 months apart), it would have been ideal if values were obtained on the same date as the lipid profile. Given our focus on the VAMC population, our demographics were skewed towards a predominantly older, male population. As the ASCVD 10-year risk calculation requires that the patient's age be known, with a lower age limit of 40, any patient younger than this age was imputed to age 40, which can lead to a left censoring bias effect. This is a limitation inherent to this risk assessment tool in general, but highlights the fact that better tools are needed in higher risk, younger aged patient populations, including spondyloarthritis with axial or peripheral disease.

We also attempted to eliminate inclusion of data from nonspondyloarthritis patients by strictly applying the ASAS criteria, although the fact that we excluded nearly half of ICD diagnoses of AS may have introduced a “selection” bias. Notably, as the patients included in our study received the majority, if not all of their medical care at the VAMC, we were able to capture all of their medical records and laboratory testing, providing detailed clarity of the clinical parameters of interest. For example, hypertension treatment, statin therapy, and DM status were readily confirmed by reviewing the patients' medical chart for time and duration of antihypertensive therapy, statin therapy, and/or supportive laboratories (e.g., HgbA1c value of >6.5%).

In conclusion, traditional CVD risk factors, as represented by the ASCVD 10-year risk score, correlated with RDW and albumin in this spondyloarthritis cohort, and RDW was also associated with age, albumin, hemoglobin, race, HLAB27 status, CRP and statin treatment. Additionally, RDW was relatively stable over time, after starting therapy. In combination with larger nonautoimmune disease cohort data, these findings support further investigation of relationships between RDW, albumin, ALC, ASCVD 10-year risk score, and CVD risk in a larger cohort of spondyloarthritis patients.

## Figures and Tables

**Table 1:** Characteristics of patient population.

Age (Years) median, (range)	56, (27-78)
Male, number (%)	42 (97.7%)
Female, number (%)	1 (2.3%)
Race, number (%)	
African American	7 (16.2%)
Caucasian	35 (81.4%)
Other	1 (2.3%)
HLA-B27 positivity, number (%)	32 (74.4%)
Inflammatory Bowel Disease, number (%)	6 (14%)
Psoriasis, number (%)	1 (2%)
Iritis, number (%)	13 (30%)
Smoking, number (%)	20 (46.5%)
HTN, number (%)	14 (32.6%)
DM, number (%)	8 (18.6%)
Statin Therapy, number (%)^a^	11 (25.6%)
Total Cholesterol mg/dL (range)	174 (90-289)
HDL mg/dL (range)	42 (23-95)
History of hyperlipidemia, number (%)^b^	18 (41.9%)
History of CAD, number (%)^c^	4 (9.3%)
ASCVD 10-year risk score median (%), range (%)	13.1 (0.7-62.2)
RDW (%), median, range^d^	13.45, (11.9-19.35)
Hemoglobin (Hgb g/dL) median, (range)	14.25 (10.75-17.55)
Absolute Lymphocyte count cells/ul x 1000 median, (range)	1.865 (0.56-8.6)
Albumin (g/dl), median, (range)	3.8 (3-4.15)
CRP (mg/L) median, (range)	8.78 (0.4-95.4)
csDMARDs only, number (%)	5 (11.6%)
Biologic monotherapy, number (%)	24 (55.8%)
Combination therapy, number (%)	6 (14.0%)

^a^Statin therapy: based on review of medication list during the period of July 1, 2013, to July 1, 2017.

^b^Hyperlipidemia which was identified based on ICD-10 code or problem list review of lipid profiles and statin use.

^c^CAD: coronary artery disease which was identified based on ICD-9/10 code or problem list review including a diagnosis of unstable angina, myocardial infarction, and/or stroke/transient ischemic attack (TIA).

^d^Average of last 2 RDW readings.

## Data Availability

The data used to support the findings of this study are available from the corresponding author upon request.
